# Do Antiborrelial Antibodies Suggest Lyme Disease in Cuba?

**DOI:** 10.3201/eid1009.031048

**Published:** 2004-09

**Authors:** Islay Rodríguez, Carmen Fernández, Marina Cinco, Rodobaldo Pedroso, Omar Fuentes

**Affiliations:** * Institute of Tropical Medicine Pedro Kourí, Havana, Cuba;; † University of Trieste, Trieste, Italy;; ‡ Medical Assistance Center of Las Terrazas Village, Cuba

**Keywords:** letter, Lyme disease, serologic report, Borrelia burgdorferi

**To the Editor:** Lyme disease is the most common vector-borne disease in the United States and parts of Eurasia (*1*). It represents a considerable emerging infectious disease threat because of its consequences to human health and the difficulties in preventing and controlling it (*2**,**3*).

In Cuba, Lyme borreliosis has never been reported. However, in the last 20 years ixodid ticks, mainly *Amblyomma cajennenses*, have been found in the human population in the Cuban village of Las Terrazas, Pinar del Río. These ixodid bites were frequent and widespread, especially in children, many of whom were hospitalized without a confirmatory laboratory diagnosis. Affected persons had symptoms associated with Lyme disease such as erythematous macules or papules, fever, fatigue, malaise, headache, arthralgias, myalgias, meningitis, peripheral radiculoneuropathies, and myocarditis (*4*).

A Cuban researcher, a specialist in ixodid ticks, was bitten several times by the ticks; dermatologic and neurologic symptoms compatible with Lyme disease (skin lesions, hyperesthesia with loss of reflexes, loss of muscular coordination, and fecal incontinence) developed. Borreliosis was not diagnosed at this stage; the diagnosis was either myeloradiculitis or Guillain-Barré syndrome. Three years later, a serologic diagnosis of Lyme disease was made by indirect immunofluorescence in a laboratory in the Czech Republic (*5*).

During 1998, serum samples from 14 persons who lived in the village Las Terrazas and had epidemiologic and clinical evidence of Lyme disease, were studied in our laboratory. We used an immunoglobulin (Ig) G and IgM–enzyme-linked immunoabsorbent assay (ELISA) kit (Enzygnost Borreliosis, Behring, Marburg, Germany), in which each strip contained wells coated with inactivated borrelial antigen (detergent extract from strain isolate PKo [*Borrelia afzelii*]), to detect specific antibodies to *B. burgdorferi* complex. The assays were performed according to the manufacturer's instructions. In our study, five serum samples had positive IgM titers and one near the cutoff value by IgM and IgG.

ELISA has been widely used to detect antibodies to *B. burgdorferi*; however, this assay is not standardized, which results in different levels of sensitivity and specificity. False-positive results may occur, especially when serum samples are obtained from persons with other illnesses (*6*).

To study possible cross-reactions with other infectious illnesses, different serologic tests were applied to the positive serum samples by using ELISA. One sample was weakly reactive to human leptospirosis (indirect hemagglutination assay with erythrocyte-sensitive substance antigen [Labiofam, Havana, Cuba]), but no samples were reactive to syphilis (rapid plasma reagin [Imefa, Havana, Cuba] and hemagglutination of *Treponema pallidum* [Oxoid, Diagnostic Reagents, Basingstoke, UK]). No indication of other infectious diseases was found.

All serum samples positive by ELISA were also analyzed by IgG and IgM Western blotting in the spirochete laboratory at the University of Trieste, Italy. The Western blotting was performed with a protein profile from whole–cell strain PKo and by applying the criteria of positivity described by Hauser et al. (*7*). Two serum samples showed clear IgM antibody bands to 41- and 23-kDa proteins. No IgG bands were observed. This test reportedly is more sensitive than ELISA for IgM detection (*6*).

We investigated the clinical manifestations of the patients with positive Western blotting. We found that one of the patients had been bitten several times by ticks and had an erythematous rash around the different bite sites; the rashes reddened and expanded over the course of a few days, with partial central clearing. The patient also had fever, hepatosplenomegaly, adenopathies, joint pain, and some nonspecific symptoms. He was given erythromycin before the laboratory results were confirmed and had a satisfactory recovery. In similar situations, repeat testing would be highly advisable. This was the same patient with low levels of antibodies to *Leptospira*. Investigating the symptoms of the other patient was not possible.

The presence of IgM antibodies is frequently confirmed in the early stage of Lyme disease (*6*). The patient's history of being bitten by an *A. cajennenses* tick, clinical manifestations of Lyme borreliosis, and specific antibodies to *B. burgdorferi* complex suggest the diagnosis of Lyme disease.

*A. cajennenses* has not been reported as a vector for Lyme disease. However, it is very abundant and aggressive in Cuba, and bites from this species are common. The genus *Ixodes*, the main vector of *B. burgdorferi* sensu lato, has not been reported in the area of the study. Several articles describe a new species in the United States, *B. lonestari*. *B. lonestari* in *A. americanum* has been confirmed in humans with erythema migrans (*8**,**9*).

No serologic test is available for antibodies to *B. lonestari*. That we found antiborrelial-complex antibodies may suggest the presence of a new species in this antigenic complex containing cross-reactive antigens, but many other studies are necessary to confirm it. This study represents the first serologic report of antiborrelial antibodies in Cuba. It suggests that Lyme borreliosis is present and that new cases can be expected in our country. Further laboratory studies are necessary for a more accurate diagnosis of this emerging infectious disease in Cuba.
